# Deciphering cellular heterogeneity and pathway dynamics in urinary samples: a UMAP-Based approach to understanding acute kidney injury

**DOI:** 10.3389/fphar.2025.1573469

**Published:** 2025-08-18

**Authors:** Cheng Yuan, Juan Wu, Yuandi Xiang, Lihua Ni

**Affiliations:** ^1^ Department of Oncology, Yichang Central People’s Hospital and The First College of Clinical Medical Science, China Three Gorges University, Yichang, Hubei, China; ^2^ Tumor Prevention and Treatment Center of Three Gorges University and Cancer Research Institute of Three Gorges University, Yichang, Hubei, China; ^3^ Clinical Medical Research Center for Precision Diagnosis and Treatment of Lung Cancer and Management of Advanced Cancer Pain of Hubei Province, Yichang, China; ^4^ Department of Dermatology, Wuhan First Hospital, Wuhan, Hubei, China; ^5^ Department of Otolaryngology-Head and Neck Surgery, Yichang Central People’s Hospital and The First College of Clinical Medical Science, China Three Gorges University, Yichang, Hubei, China; ^6^ Department of Nephrology, Zhongnan Hospital of Wuhan University, Wuhan, Hubei, China

**Keywords:** acute kidney injury (AKI), MAPK1 (ERK1/2), single-cell RNA sequencing (scRNA-seq), urinary biomarkers, monocyte

## Abstract

**Background:**

Acute kidney injury (AKI) is characterized by rapid loss of renal function and is associated with severe clinical outcomes. Understanding the cellular heterogeneity in urine samples during AKI may provide insights into the underlying pathophysiological mechanisms and potential therapeutic targets.

**Objectives:**

To explore the cellular composition and gene expression patterns in urine samples from AKI and non-AKI conditions using Uniform Manifold Approximation and Projection (UMAP) to identify key cellular interactions and pathway activations related to AKI.

**Methods:**

We utilized publicly available the dataset GSE180595 from the Gene Expression Omnibus (GEO) database. Urine samples were collected from AKI and non-AKI patients. Single-cell RNA sequencing (scRNA-seq) was performed to profile the mononuclear cell populations. Differential gene expression analysis was conducted to identify key molecular pathways, with a focus on ECM-related pathways. MAPK1 expression was quantified and compared between the two patient groups.

**Results:**

UMAP analysis revealed significant differences in cellular composition between AKI and non-AKI samples. Fifteen unique cell clusters were identified, each associated with distinct transcriptional profiles. In AKI samples, increased clustering of immune response cells such as monocytes was observed. Pathway analysis highlighted enhanced activation of DNA replication and ECM-related genes pathways in cells from AKI conditions, indicating their potential roles in injury response and tissue remodeling. The differential gene enrichment analysis identified ECM-related pathways as significantly enriched in the AKI group, with MAPK1 being a crucial gene regulating these pathways.

**Conclusion:**

Our findings provide evidence that MAPK1 is upregulated in urinary mononuclear cells of AKI patients and plays a key role in regulating ECM-related pathways. MAPK1 could serve as a potential biomarker for AKI diagnosis and prognosis and may represent a promising therapeutic target for limiting ECM remodeling and fibrosis in AKI. Further studies are needed to explore the clinical implications of targeting MAPK1 in AKI treatment.

## Introduction

Acute Kidney Injury (AKI) is a clinical syndrome characterized by a rapid decline in kidney function, which can result from a variety of causes, including ischemia, nephrotoxicity, infection, and sepsis (Al-Jaghbeer et al., 2018; Sun et al., 2023; Seo et al., 2024). It is associated with high morbidity and mortality rates, particularly in critically ill patients, and often leads to the development of chronic kidney disease (CKD) if not managed appropriately (Ogurt et al., 2022; Noble et al., 2020). Despite advancements in medical interventions, early detection and accurate assessment of the severity of AKI remain significant challenges in clinical practice (Li et al., 2024). Current diagnostic methods mainly rely on serum creatinine levels and urine output, both of which are delayed markers and do not capture the underlying cellular mechanisms of AKI (Kellum et al., 2021). Therefore, there is a pressing need for more sensitive and specific tools to diagnose AKI at earlier stages and understand the pathophysiological processes driving its progression.

Urinary samples, which are readily available and non-invasive, offer a valuable source of information for studying kidney injury (Baek et al., 2025; Waikar et al., 2025). During AKI, a complex array of cellular events occurs in the kidney, including damage to renal tubular cells, infiltration by immune cells, and remodeling of the extracellular matrix (Charkviani et al., 2025; Chen et al., 2024). However, these cellular changes are not easily detectable using conventional methods such as histology or bulk transcriptomics. To fully understand the cellular diversity within the kidney and the molecular pathways driving AKI, a more refined analysis is needed.

Single-cell RNA sequencing (scRNA-seq) has emerged as a powerful tool for interrogating cellular heterogeneity and gene expression dynamics at an unprecedented resolution (Ding et al., 2020). This technique provides detailed insights into individual cell types, their functional states, and their interactions during disease (Andrews et al., 2021). However, applying scRNA-seq to kidney tissue is challenging due to the complexity and tissue-specific features of the kidney. Urine, which contains a mixture of shed epithelial cells, immune cells, and extracellular vesicles, offers a less invasive and more accessible alternative for studying kidney injury at the cellular level.

In this study, we leverage the power of single-cell transcriptomics applied to urinary samples to examine the cellular diversity and dynamic gene expression changes that occur during AKI. To effectively analyze the high-dimensional data generated by scRNA-seq, we utilize Uniform Manifold Approximation and Projection (UMAP), a nonlinear dimensionality reduction technique that excels in preserving local and global structure while visualizing complex data. UMAP enables the identification of distinct cell clusters, highlighting their gene expression profiles, and provides a means to uncover the cellular mechanisms driving AKI progression.

## Materials and methods

### Data source

To explore cellular heterogeneity and pathway dynamics in urinary samples from AKI patients, we utilized publicly available data from the Gene Expression Omnibus (GEO) database. Specifically, we obtained the dataset GSE180595, which contains high-resolution transcriptomic profiles of urinary cells from individuals diagnosed with or without AKI. AKI cases were identified using the KDIGO (Kidney Disease: Improving Global Outcomes) diagnostic criteria, defined as a rise in serum creatinine (sCr) > 0.3 mg/dL within 48 h or >1.5 × baseline sCr. Non-AKI controls were selected based on age- and sex-matching, with no significant change in sCr during hospitalization. This dataset includes scRNA-seq data, which provides an in-depth analysis of gene expression at the single-cell level, offering a valuable resource for investigating the molecular mechanisms underlying AKI.

### Data preprocessing

The raw data for GSE180595 was downloaded from the GEO database in the form of gene expression counts and associated metadata. We performed the following preprocessing steps to prepare the data for downstream analysis.

#### Quality control (QC)

We first removed low-quality cells based on standard QC metrics, such as the number of detected genes per cell, the percentage of mitochondrial genes, and the number of total unique molecular identifiers (UMIs) per cell. Cells with fewer than 200 detected genes or with greater than 10% mitochondrial gene expression were discarded to ensure only high-quality cells were retained for analysis.

#### Normalization

After QC, the expression matrix was normalized to account for differences in sequencing depth across cells. The data was normalized using a global-scaling method called log-normalization, which scales the gene expression to counts per million (CPM) and applies a logarithmic transformation to stabilize the variance.

#### Filtering of lowly expressed genes

Genes that were expressed in fewer than 5% of cells were removed to reduce noise and computational burden.

### Dimensionality reduction and UMAP analysis

To investigate the cellular heterogeneity in the urinary samples from AKI patients, we employed UMAP, a powerful technique for visualizing high-dimensional single-cell data. The following steps were taken for UMAP-based analysis.

#### Principal component analysis (PCA)

After normalization and filtering, we performed PCA to reduce the dimensionality of the dataset. The first 20 principal components (PCs) were selected for downstream analysis, based on the scree plot and explained variance ratio.

#### Cell clustering

We used the Seurat R package (version 4.0) to cluster cells based on their gene expression profiles. The clustering algorithm applied was the Louvain algorithm, which groups cells based on shared gene expression patterns. The resolution parameter was set to 0.5 to ensure meaningful clustering while maintaining the granularity of cell subpopulations.

#### UMAP embedding

To visualize the cellular heterogeneity, we performed UMAP using the top principal components from PCA. The UMAP projection was created to map the high-dimensional gene expression data into a 2D space, facilitating the identification of distinct cell clusters (n_neighbors = 20, min_dist = 0.5). These clusters represent different cell types or functional states that are enriched in specific gene expression signatures.

### Identification of cell types and pathways

#### Cell type annotation

Once the cell clusters were identified through UMAP, we performed cell type annotation by comparing the cluster-specific gene expression signatures to known marker genes available in the literature. Specifically, we used marker genes for renal tubular cells, immune cells (such as macrophages and T-cells), endothelial cells, and fibroblasts to classify the major cell populations present in the urinary samples.

#### Differential gene expression analysis

We conducted differential gene expression analysis between different AKI severity groups and healthy controls, using the DESeq2 package. This analysis aimed to identify genes that were significantly upregulated or downregulated in response to AKI. We then performed pathway enrichment analysis using the ClusterProfiler R package to identify key biological pathways altered in AKI.

#### Pathway analysis

To identify the specific signaling pathways involved in AKI, we utilized the pathway database to perform pathway enrichment analysis on the differentially expressed genes. Significant pathways were considered those with a false discovery rate (FDR) < 0.05.

### Statistical analysis

Differential expression was determined using DESeq2, with a significance threshold of adjusted p-value (FDR) < 0.05. For the UMAP visualizations, we used the Seurat package in R. Statistical significance in pathway enrichment analysis was determined using a hypergeometric test with a false discovery rate threshold of 0.05.

## Results

### Analysis of cellular RNA and mitochondrial content across distinct cell identities

**FIGURE 1:**
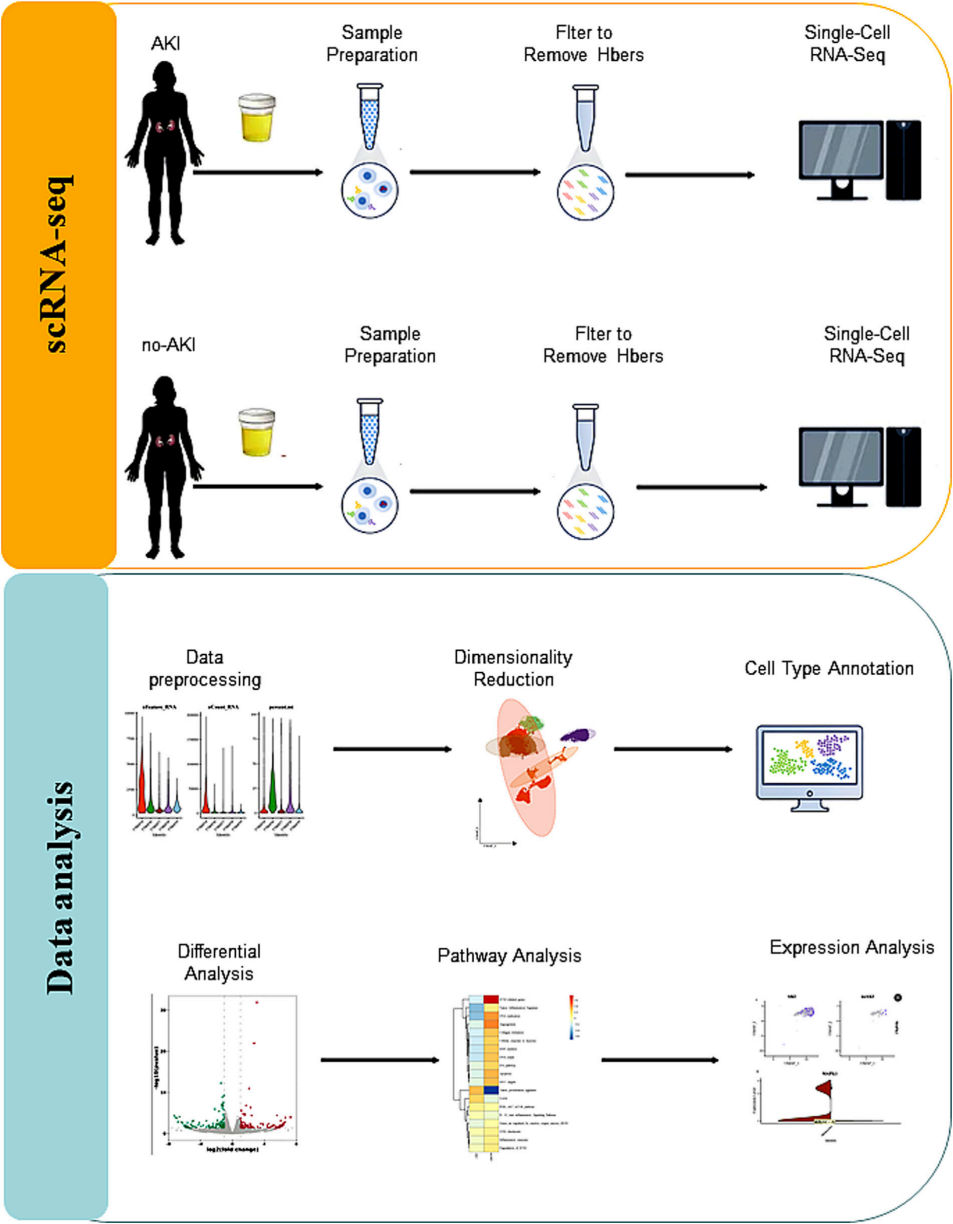
Single-cell RNA sequencing analysis of urine from AKI and non-AKI patients. This flowchart illustrates the process of single-cell RNA sequencing (scRNA-seq) analysis of urine samples from patients with Acute Kidney Injury (AKI) and non-AKI individuals. The process begins with patient selection and urine sample collection, followed by single-cell isolation. RNA is extracted from the isolated cells, and high-throughput scRNA-seq is performed. The data undergo preprocessing, including quality control and normalization. Cellular subpopulations are identified through clustering analysis, and differential gene expression is evaluated between AKI and non-AKI patients. Gene Set Variation Analysis is performed to identify pathways associated with AKI. The results are visualized using heatmaps, PCA, and UMAP, with the final analysis aiming to provide insights into the molecular mechanisms of AKI.

**FIGURE 2:**
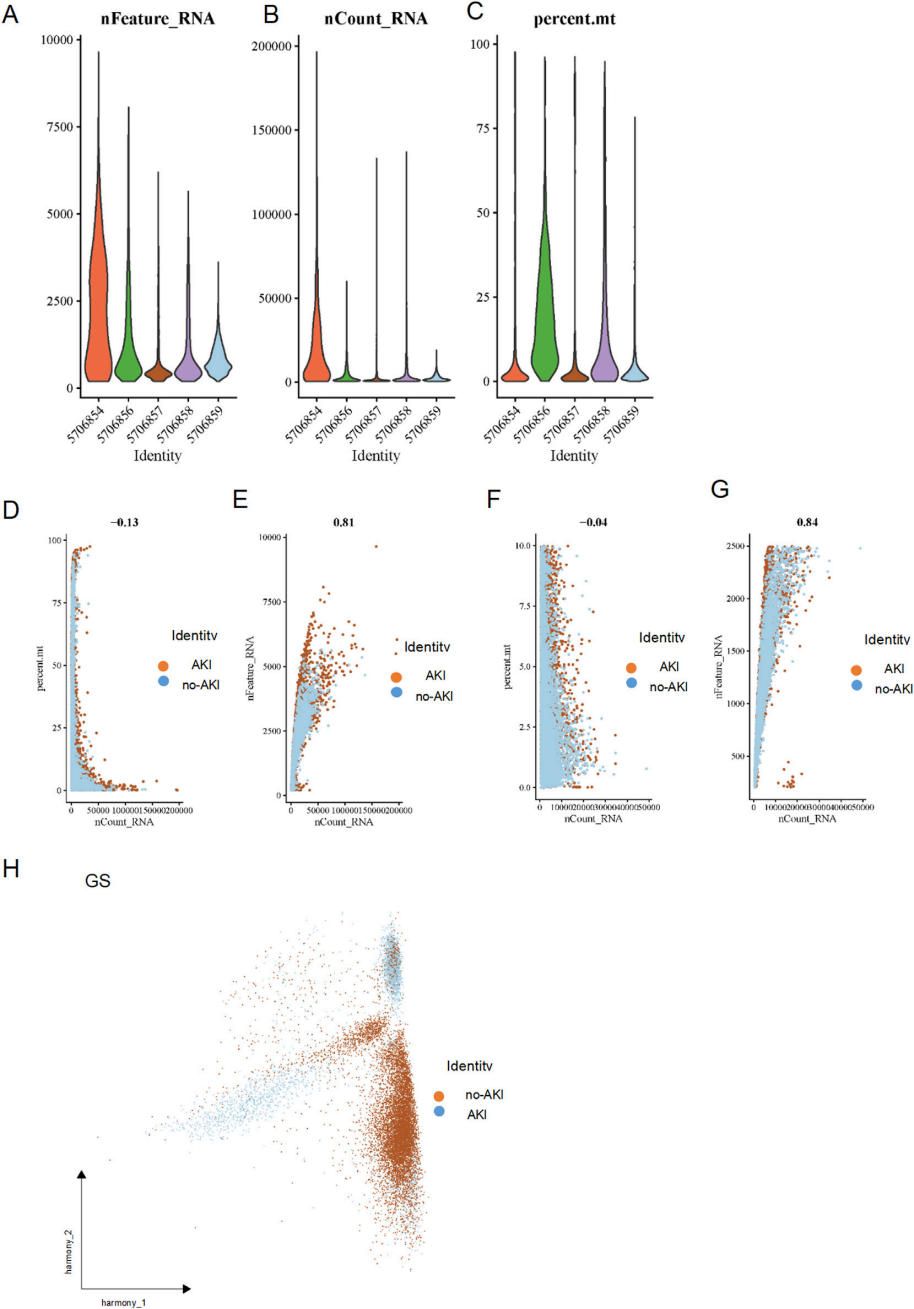
Comprehensive Analysis of RNA and Mitochondrial Content Variability Across Different Cell Identities. **(A)** nFeature_RNA: Violin plots showing the distribution of RNA features across different cell identities. **(B)** nCount_RNA: Violin plots depicting total RNA count distribution across various cell. **(C)** percent. mt: Violin plots representing the percentage of mitochondrial RNA content in different cell. **(D)** Scatter plot of nCount_RNA vs percent before filtering. mt: The scatter plot showing the relationship between total RNA count (nCount_RNA) and mitochondrial RNA percentage (percent.mt) with a correlation coefficient of −0.13. **(E)** Sequencing depth before filtering. **(F)** Scatter plot of nCount_RNA vs percent after filtering. mt: This plot illustrates another view of the relationship between RNA counts and mitochondrial percentage with a correlation coefficient of −0.04. **(G)** Sequencing depth after filtering. **(H)** The multiple gene populations with large differences obtained after PCA analysis.

The overall workflow of our study is presented in Figure 1. Comprehensive single-cell RNA sequencing was employed to elucidate the diversity in RNA expression profiles and mitochondrial content across various identified cell populations. Violin plots revealed a wide range of RNA features and counts, distinguishing the cell identities (Figures 2A,B).

Moreover, the analysis of mitochondrial RNA percentage showcased notable variability across the different cell identities, with some cells exhibiting significantly higher mitochondrial content, which could be indicative of altered metabolic states or stress responses (Figure 2C). Correlation analysis further demonstrated varying relationships between total RNA count and mitochondrial content, with correlation coefficients ranging from −0.13 to −0.04 after filtering, and the sequencing depth was 0.81 and 0.84. (Figures 2D–G). The PCA analysis (Figure 2H) successfully segregated the cells into two distinct clusters, highlighting the heterogeneity within the sampled population and potentially reflecting different functional states or lineage commitments within the urine specimen under study.

These findings underscore the complex interplay between RNA expression levels and mitochondrial dynamics, which may influence cellular function and phenotype in renal pathophysiology. This granular analysis provides a deeper understanding of the cellular landscapes, essential for unraveling the molecular mechanisms underlying renal disorders.

### Comprehensive insights into gene expression variability and cellular dynamics

**FIGURE 3:**
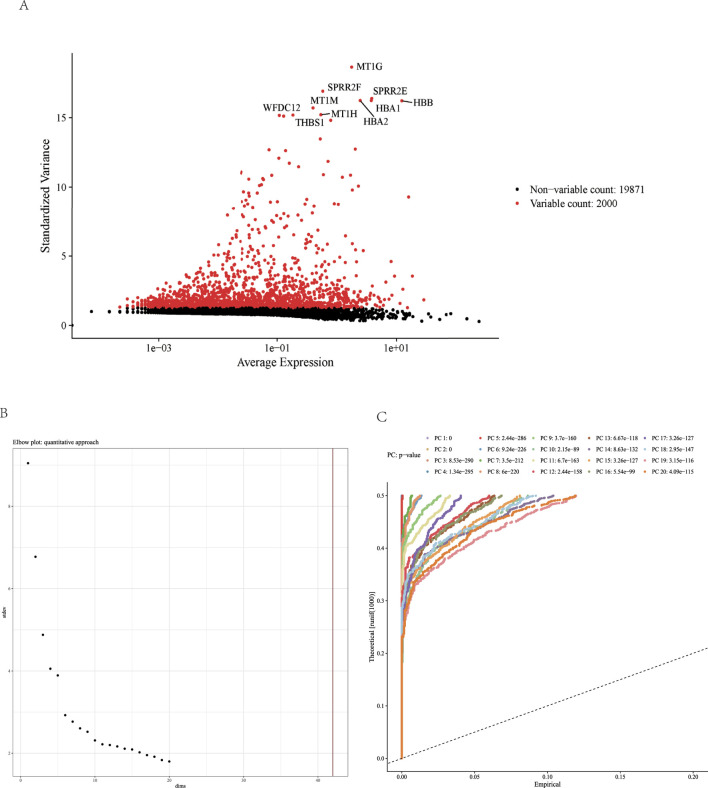
Gene Expression Variability and Its Impact on Gene Regulation. **(A)** Scatter plot displaying standardized variance versus average expression levels of genes in a given dataset. Each point represents a gene; red points indicate genes with high variability (total count: 2000), while black points represent genes with low or non-variable expression (total count: 19871). Key highly variable genes are labeled, illustrating their potential regulatory significance in the dataset. **(B)** Use the ElbowPlot function to evaluate the PC. **(C)** The visualization result of the JackStrawPlot function.

Our analysis delved into the variability and average expression levels across a broad spectrum of genes within diverse cell populations. Figure 3A illustrates this relationship, highlighting a subset of genes represented by red dots, which demonstrated significantly higher variability in expression. Notable among these, genes such as MTIG, SPRR2F, and HBA1 appear to play pivotal roles in cellular regulation, possibly due to their dynamic expression patterns across different cellular states or in response to environmental stimuli.

The expression landscape, as shown in Figure 3B, reveals a predominant trend of sparse expression across the majority of genes, interspersed with occasional peaks in higher quantiles. This distribution suggests most of the real signals are captured in the 20 principal components (PCs).

Further illustrating the biological impact of these expression patterns, Figure 3C presents Significance results of PCs for various gene sets. This relationship underscores the potential of gene expression levels to serve as the p-value distribution of each PCs with the uniform distribution.

Together, the depicted data weave a complex narrative of gene expression variability, emphasizing its significance in shaping cellular behavior and influencing clinical outcomes in kidney diseases.

### Differential cellular composition and interaction in AKI and non-AKI conditions revealed by UMAP analysis

**FIGURE 4:**
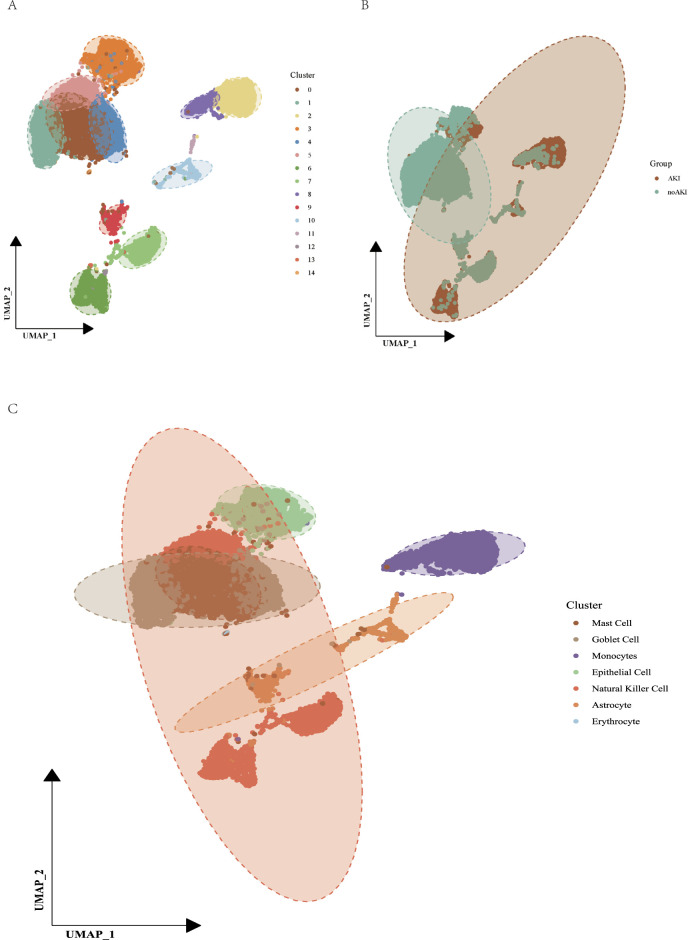
Multi-panel Uniform Manifold Approximation and Projection (UMAP) analysis illustrating cellular distribution and pathway activation. **(A)** UMAP displaying the clustering of various cell types within urine samples. Each cluster is color-coded to represent different cell types as indicated in the legend on the right, with a total of 15 clusters identified. This panel underscores the heterogeneity of cell populations in urine, highlighting their diverse transcriptional profiles. **(B)** UMAP visualization segregating cells from acute kidney injury (AKI) conditions (red) and non-acute kidney injury (non-AKI) conditions (green). Dashed outlines indicate regions of overlap and distinct clustering, suggesting differential cellular states or interactions in response to kidney injury. **(C)** Focused UMAP analysis on specific cellular clusters including Mast Cells, Goblet Cells, Monocytes, and other identified types, each mapped in distinct colors. Overlaying dashed lines delineate significant overlaps between clusters, providing insights into the interplay of different cell types and their potential roles in disease pathology and recovery processes.

Our comprehensive UMAP analysis elucidated distinct cellular landscapes in urine samples from patients with and without AKI, as depicted in Figure 4. Figure 4A illustrates a diverse array of cell types across all samples, with 15 unique clusters identified, each denoted by a distinct color. This diversity highlights the complex cellular makeup in urine, suggesting a wide range of cellular responses and states within these samples.

In Figure 4B, UMAP analysis distinctly separated cells derived from AKI conditions (colored in red) from those in non-AKI states (colored in green), with the outlined areas indicating significant overlap. This overlap not only underpins the transitional nature of certain cellular states associated with kidney injury but also indicates shared cellular pathways that are potentially active in both health and disease states.

Further detailed in Figure 4C, specific cell types such as Mast Cells, Goblet Cells, Monocytes, and others are clearly segregated into defined clusters, yet display interactions as suggested by the overlapping regions. This pattern reflects the specialized roles these cells may play in the pathophysiology of kidney injury, potentially driving or responding to the inflammatory and reparative processes inherent to AKI.

These findings not only advance our understanding of the cellular basis of kidney injury but also provide a framework for identifying potential biomarkers and therapeutic targets in AKI. The data demonstrate the utility of UMAP in capturing the intricate cellular dynamics within urine, offering a nuanced view of the cellular activities during kidney injury and recovery.

### Cellular composition and gene expression profiling in AKI versus Non-AKI conditions

**FIGURE 5:**
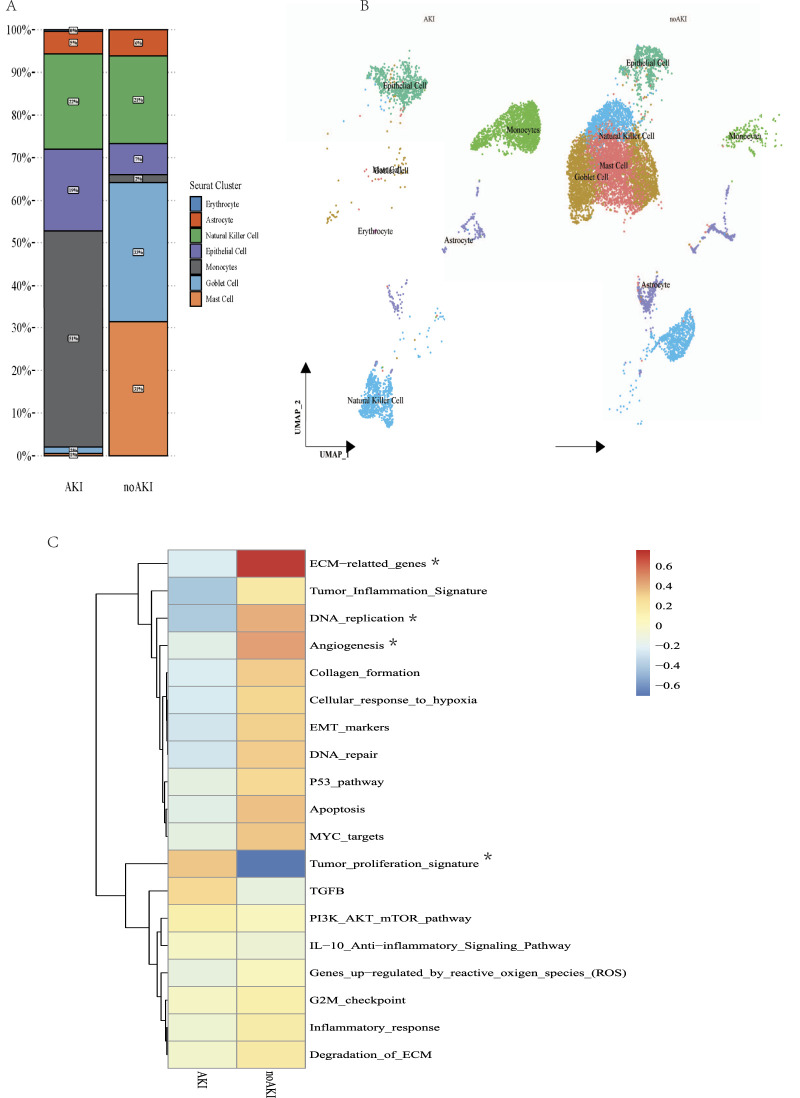
Comparative Analysis of Cellular and Molecular Responses in AKI and non-AKI Conditions. **(A)** Stacked bar graph displaying the proportion of various cell types in urine samples from acute kidney injury (AKI) and non-acute kidney injury (non-AKI) conditions. Each cell type is color-coded for clarity, illustrating significant differences in cellular composition between conditions. **(B)** UMAP plots showing the distribution of cell types in AKI and non-AKI samples. Each dot represents a single cell, colored according to the cell type designation shown in panel. **(C)** Heatmap of gene expression profiling related to various biological pathways and processes in cells from AKI and non-AKI conditions. The heatmap colors represent z-score normalized expression levels, ranging from low (blue) to high (red) *FDR <0.05.

Our analysis using UMAP coupled with detailed cellular and molecular profiling revealed significant differences in the cellular composition and gene expression patterns between AKI and non-AKI conditions. As depicted in Figure 5A, the distribution of cell types within urine samples from AKI patients showed a distinct increase in specific cells such as lymphocytes and natural killer cells, contrasted with a diverse cellular environment in non-AKI conditions. This variation highlights the specific cellular responses elicited by kidney injury.

Figure 5B further illustrates the spatial segregation of cell types in AKI versus non-AKI samples. The UMAP plots clearly demonstrate unique clustering of cell types, which are color-coded to match the identity established in Figure 5A. These clusters indicate not only the presence of distinct cellular populations in response to injury but also suggest potential interactions and signaling pathways that may be disrupted or activated in AKI.

Moreover, Figure 5C presents a heatmap of gene expression profiling for a range of biological pathways, emphasizing alterations in the cellular machinery during AKI. Notable pathways such as DNA replication and ECM-related genes exhibited marked differences in expression levels. This heatmap elucidates the underlying molecular mechanisms that might contribute to the pathology and subsequent recovery in AKI, providing insights that could be pivotal for therapeutic intervention.

Together, these analyses underscore the profound impact of acute kidney injury on the cellular and molecular landscape, revealing how shifts in cellular composition and gene expression can provide insights into the mechanisms of injury and potential targets for treatment. This comprehensive cellular and molecular characterization enhances our understanding of the dynamic changes occurring in kidney tissues during injury, offering a valuable resource for developing more effective diagnostic and therapeutic strategies.

### MAPK1 expression analysis

The enrichment analysis of monocyte in the urine of both patient groups indicated significant involvement of the ECM - related pathway. Based on the differential gene expression levels and the statistical significance of the observed differences, we hypothesize that MAPK1 might be a core gene regulating the ECM-related pathways within the monocyte cells in the urine of these two patient groups.

**FIGURE 6:**
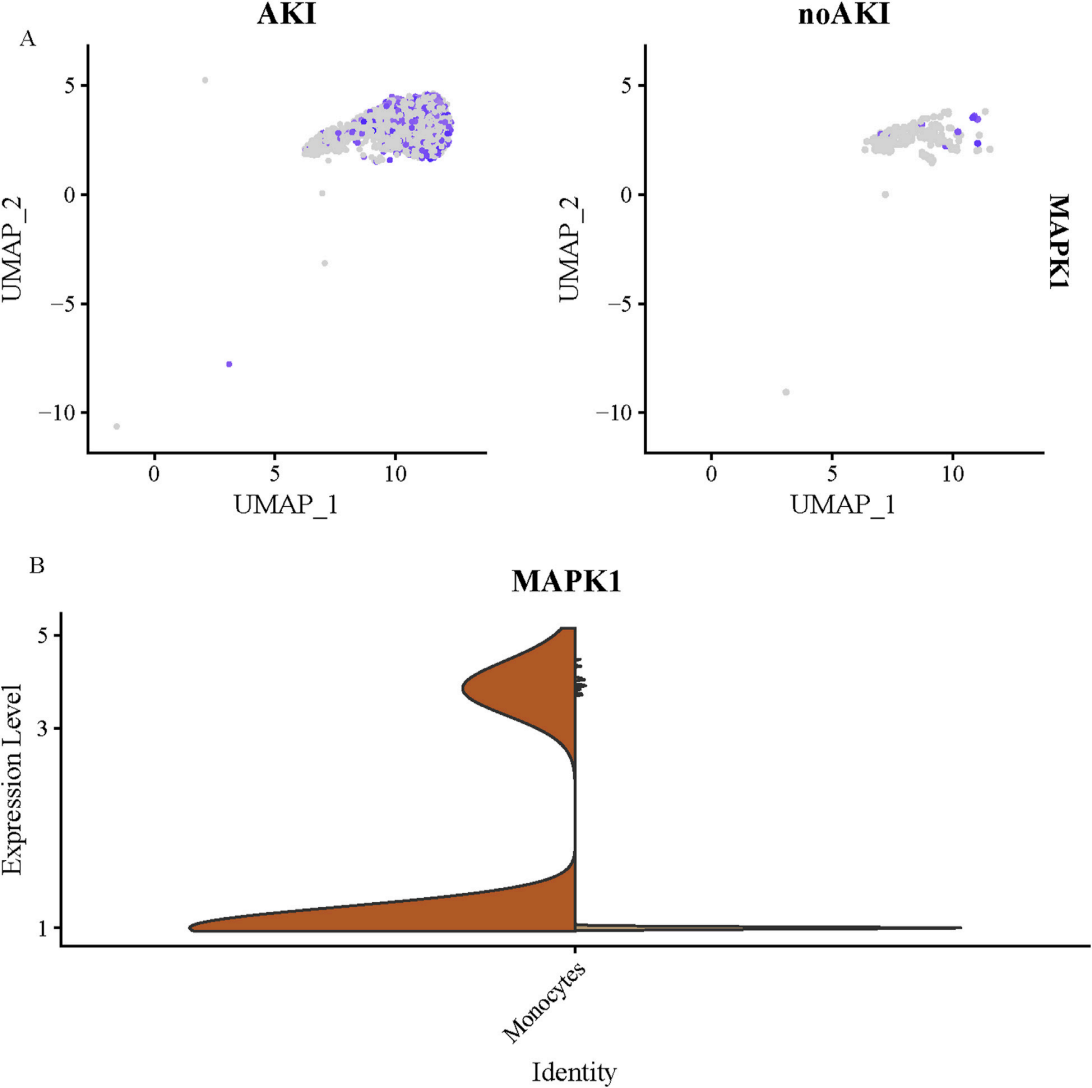
UMAP Analysis and MAPK1 Expression in Urinary monocyte from AKI and Non-AKI Patients. **(A)** UMAP analysis of single-cell RNA sequencing data from urinary monocyte of AKI and non-AKI patients. **(B)** Quantification of MAPK1 expression in urinary monocyte cells. MAPK1 expression levels were significantly higher in the AKI group compared to the non-AKI group (FDR <0.05). The bar graph shows the relative expression of MAPK1 in cells from both groups, with AKI patients exhibiting a marked increase in MAPK1 expression. The data suggest that MAPK1 plays a key role in the regulation of ECM-related pathways in the monocyte cells from AKI patients.

The UMAP plot (Figure 6A) clearly distinguishes between the two patient groups, AKI and non-AKI. In the UMAP_1 vs. UMAP_2 plot, cells from AKI patients form a distinct cluster that was separated from the non-AKI cell population. The AKI cell clusters showed a broader spread across the UMAP_1 axis, suggesting increased cellular diversity or altered cell states in response to kidney injury. In contrast, the non-AKI patient cells clustered more tightly along the UMAP_1 axis, indicating a less heterogeneous cell population. This suggests a relatively stable cellular profile in the absence of kidney injury. The separation between the AKI and non-AKI groups in the UMAP plot emphasizes the distinct molecular signatures between the two groups. The distribution of specific cell type also varied between the two groups, with the AKI group showing an increased proportion of monocyte, which may be related to the inflammatory response and tissue injury in AKI.

To further investigate the molecular changes in monocyte from AKI and non-AKI patients, we quantified the expression levels of MAPK1 in these cells. The MAPK1 expression levels in monocyte from the AKI group were significantly higher compared to the non-AKI group (FDR <0.05, Figure 6B). This suggests that MAPK1 is actively involved in regulating key cellular processes such as inflammation, apoptosis, and stress response within monocyte during AKI.

## Discussion

This study investigates the role of MAPK1 (ERK1/2) in the pathogenesis of AKI by analyzing its expression in urinary monocyte from AKI and non-AKI patients. Our results demonstrated a significant upregulation of MAPK1 in the AKI group compared to the non-AKI group, suggesting its involvement in regulating ECM -related pathways and inflammation during kidney injury.

### MAPK1 (ERK1/2) in AKI pathogenesis

MAPK1, a key component of the extracellular signal-regulated kinase (ERK) pathway, is involved in a variety of cellular processes including proliferation, differentiation, survival, and response to stress (Lin et al., 2024; Quan et al., 2024). Our findings confirmed that MAPK1 was upregulated in monocyte of AKI patients, supporting the hypothesis that MAPK1 activation plays a central role in mediating the inflammatory response and ECM remodeling during AKI. This was consistent with previous studies demonstrating that ERK1/2 signaling is activated in response to cellular stress and is involved in the regulation of inflammatory cytokines, cellular apoptosis, and tissue repair (Hu et al., 2016; Lee et al., 2016; Muscella et al., 2024; Jiang et al., 2025).

In AKI, the activation of MAPK1 was likely a consequence of the kidney’s response to injury, promoting the release of pro-inflammatory mediators and contributing to renal damage (Ajay et al., 2024; Wang et al., 2023). MAPK1 has been shown to modulate the expression of several ECM components, including collagen and fibronectin, through the activation of downstream signaling molecules such as c-Fos and c-Jun (Li et al., 2022; Jia et al., 2025). The upregulation of MAPK1 in AKI patients, as observed in our study, might thus be involved in the excessive ECM remodeling that was characteristic of AKI and subsequent fibrosis.

### Differential ECM remodeling in AKI vs. Non-AKI

Our differential gene expression analysis revealed significant enrichment of ECM-related pathways in the monocyte from AKI patients. ECM remodeling was a critical process in the pathophysiology of AKI, as it impacts both the structural integrity and function of renal tissue (Gupta et al., 2024; Zhang et al., 2024; Lee et al., 2011). ECM components such as collagen, laminin, and fibronectin are critical for maintaining tissue architecture, but their dysregulation can lead to fibrosis and impaired tissue repair. MAPK1, through its regulation of ECM-related genes, may play a key role in the excessive ECM deposition and fibrosis that occurs in AKI (Blache et al., 2021). The relationship between MAPK1 and ECM components in AKI could provide valuable insights into potential therapeutic targets to limit fibrosis and promote recovery.

### MAPK1 as a potential biomarker and therapeutic target

The upregulation of MAPK1 in urinary monocyte of AKI patients highlights its potential as a diagnostic and prognostic biomarker. Given that MAPK1 can be detected in urine-derived monocyte cells, this non-invasive approach could provide an early indicator of kidney injury, complementing traditional biomarkers such as serum creatinine and urine output. Early detection of MAPK1 activation could help identify patients at high risk of progression to more severe AKI or CKD. The sensitivity, specificity, and predictive value of MAPK1 as a biomarker have not yet been thoroughly evaluated. Additionally, comparisons of MAPK1’s performance with established clinical biomarkers, such as serum creatinine, remain to be performed. Future prospective clinical studies are warranted to systematically evaluate the diagnostic accuracy and clinical utility of MAPK1, particularly in comparison to traditional markers. Such validation could confirm whether MAPK1 provides superior or complementary clinical information and clarify its precise role and practical advantages in clinical settings.

Additionally, targeting MAPK1 signaling could offer a novel therapeutic strategy in AKI management. Inhibition of MAPK1 signaling has been shown to reduce inflammation, fibrosis, and ECM remodeling in experimental models of kidney injury (Jiang et al., 2020; Yin et al., 2022). Therapeutically, targeting the MAPK1 pathway has already shown promise in preclinical models. Inhibitors such as PHPS1 and U0126 have demonstrated renoprotective effects by suppressing ERK phosphorylation, thereby reducing tubular apoptosis, oxidative stress, and inflammatory cytokine release (Liu et al., 2025; Wang et al., 2025). These findings indicate that MAPK1 inhibition could be a feasible therapeutic strategy, especially in AKI subtypes characterized by maladaptive ERK activation.

## Limitations and future directions

While our study provides compelling evidence for the role of MAPK1 in AKI, several limitations should be considered. First, the cross-sectional nature of our study limits our ability to establish a causal relationship between MAPK1 activation and kidney injury. Longitudinal studies tracking MAPK1 expression over time would provide more insights into its temporal role in AKI development and recovery. Second, while we focused on monocyte cells in urine, future studies could investigate the role of MAPK1 in other cell types within the kidney, such as proximal tubular cells, endothelial cells, and fibroblasts, which may contribute to ECM remodeling and fibrosis in AKI. Further investigation into the downstream effectors of MAPK1 in kidney cells will be essential to fully understand its role in ECM regulation. Finally, this study relies on publicly available data, which contains urine-derived cells from a small cohort: AKI patients (n = 5) and non-AKI controls (n = 3). While urine samples offer a non-invasive and accessible window into renal injury processes, they may not fully capture the complex spatial microenvironment and cellular heterogeneity of kidney tissue itself. As such, transcriptomic profiles derived from urine cells may reflect a partial view of intrarenal dynamics.

## Conclusion

In conclusion, our study provides strong evidence that MAPK1 is upregulated in monocyte cells from AKI patients and is likely involved in regulating ECM-related pathways during kidney injury. This upregulation of MAPK1 is associated with the severity of kidney dysfunction and may contribute to pathological ECM remodeling and fibrosis. MAPK1 could serve as a potential biomarker for AKI and a therapeutic target for preventing renal fibrosis and promoting recovery. Further research is needed to validate these findings and explore the therapeutic potential of MAPK1 inhibition in AKI treatment.

## Data Availability

The datasets presented in this study can be found in online repositories. The names of the repository/repositories and accession number(s) can be found in the article/supplementary material.
